# First report of mobile colistin resistance gene *mcr*-1 in avian pathogenic *Escherichia coli* isolated from turkeys in the Gaza Strip, Palestine

**DOI:** 10.14202/vetworld.2023.1260-1265

**Published:** 2023-06-08

**Authors:** Ahmed M. Thabet, Ibrahim M. Alzuheir, Nahed A. Al Laham, Belal Y. Abu Helal, Adnan F. Fayyad, Nasr H. Jalboush, Mohammad H. Gharaibeh

**Affiliations:** 1 Thabet Center for Veterinary Services diagnostic laboratory, Gaza Strip, Palestine; 2 Department of Veterinary Medicine, Faculty of Agriculture and Veterinary Medicine, Al-Azhar University, Gaza Strip, Palestine; 3 Department of Veterinary Medicine, Faculty of Agriculture and Veterinary Medicine, An-Najah National University, P.O. Box 7 Nablus, Palestine; 4 Department of Laboratory Medicine, Faculty of Applied Medical Sciences, Al-Azhar University, Gaza Strip, Palestine; 5 Department of Basic Veterinary Medical Science, Faculty of Veterinary Medicine, Jordan University of Science and Technology, P. O. Box 3030 Irbid, 22110, Jordan

**Keywords:** avian pathogenic *Escherichia coli*, colistin, Gaza Strip, turkey

## Abstract

**Background and Aim:**

Colistin is used to treat avian pathogenic *Escherichia coli* (APEC), a microorganism that affects turkey meat production in the Gaza Strip and worldwide. However, the recent emergence of plasmid-borne mobile colistin resistance (*mcr*) genes in pathogenic *E. coli* strains is a serious antimicrobial resistance (AMR) challenge for both human and animal health. In December 2018, colistin was banned as a veterinary antimicrobial in the Gaza Strip. This study aimed to detect and track the prevalence of colistin-resistant APEC isolated from turkey flocks in the Gaza Strip.

**Materials and Methods:**

This study investigated 239 APEC isolates from turkey flocks in the Gaza Strip between October 2018 and December 2021 (at 6-month intervals). The colistin-resistant APEC strains were detected using the broth microdilution method. The *mcr-1* gene was identified using a polymerase chain reaction.

**Results:**

The overall colistin resistance among the isolated APECs was 32.2% during the study period. The average resistance in the first interval was 37.5%, which significantly decreased to 9.3% in the last interval. Among the 77 phenotypically resistant isolates, 32.4% were positive for *mcr-1*. The average abundance of *mcr*-1 in the first interval was 66.6%, which decreased to 25% in the last interval.

**Conclusion:**

To the best of our knowledge, this is the first study reporting the presence of the *mcr-1* gene among the APEC isolates from turkeys in the Gaza Strip. Banned veterinary use of colistin significantly decreased the percentage of resistant APEC isolates from turkeys in Gaza Strip. Further studies are needed to investigate other colistin resistance genes and track the emergence of AMR.

## Introduction

Colistin is a cationic polypeptide antimicrobial agent that affects the outer membrane of Gram-negative bacteria [1]. Previously, it was commonly used to treat turkey flocks [2]. However, the rapid spread of bacteria resistant to antimicrobials, such as colistin, is a global public health concern. Colistin resistance is generated through chromosome- or plasmid-mediated genes [3]. Plasmid-mediated colistin resistance was first characterized in 2015 through the identification of the mobile colistin resistance gene (*mcr-1)* in *Escherichia coli* from animals and humans in China [4]. Shortly after this, the *mcr-1* gene was detected in several other pathogens [5]. To date, at least 10 *mcr* genes have been identified in bacteria isolated from the environment, plants, animals, and humans [6]. Of these, five variants were identified in *Enterobacteriaceae* worldwide [7]. Among all the identified gene variants, *mcr-1* is the most widely distributed gene globally [8].

Turkey’s meat production has grown in the Gaza Strip in the last decade, with a production capacity of more than one million birds per year [9]. Free-range turkey farming with high-density raising and insufficient biosecurity is the most common rearing method in the Gaza Strip, rendering the birds more susceptible to environmentally conditioned diseases and requiring antimicrobial treatment, and promoting antimicrobial resistance (AMR). Avian pathogenic *Escherichia coli* (APEC) causes devastating economic losses in poultry industries in developing countries [10]. Colistin is widely used to treat APEC in poultry as a primary or secondary pathogen [11–13]. Over 60% of all antibiotics are used in animal production [14]. The widespread occurrence of APEC in turkeys’ meat has prompted farmers to use parenteral antimicrobial agents. Moreover, the misuse of antimicrobials against APEC significantly compromises the efficacy of most antimicrobials. This resistance can be transferred to humans and other livestock due to genetic and phenotypic AMR [15–17]. In 2016, the World Health Organization listed colistin as an important antibiotic for treating humans infected with multidrug-resistant Gram-negative bacteria [18]. In December 2018, all veterinary products containing colistin were banned in the Gaza Strip to control the development of AMR against colistin, a last-resort antibiotic in human medicine.

The presence of bacterial strains harboring *mcr* in poultry meat consumed by humans is a critical issue as it causes serious bacterial diseases with no treatment options. Therefore, further studies are needed to determine the presence of colistin-resistant APECs in Palestinian meat products.

This study aimed to assess the prevalence of colistin resistance in APEC using broth microdilution. The presence of *mcr-1* was investigated using polymerase chain reaction (PCR). Further, to elucidate the effects of the colistin ban on the prevalence of *mcr-1* among APECs isolated from clinical samples, the study period ranged from October 2018 to December 2021.

## Materials and Methods

### Ethical approval

Samples were obtained from cases sent to the Thabet Center for Veterinary Services (TCVS) Diagnostic Laboratory, Gaza Strip-Palestine, to diagnose the disease under the usual veterinary service work in Palestine. In addition, swabs were collected from dead birds only. Therefore, ethical approval was not necessary for this study.

### Study period and location

The study was conducted from October 2018 to December 2021. A total of 239 suspected APEC were isolated from turkey flock cases presented at TCVS for the diagnosis. The clinical findings and postmortem lesions were recorded. Sterile transport swabs (APTATA^®^, Canelli, Italy) were used to obtain samples aseptically from the lesions of each dissected bird. The geographical location of the farm (the five main regions in Gaza Strip (North, Gaza, Middle, Khan Younes, and Rafah), the age of the birds, and the date of sampling were recorded for each sample.

### Identification and confirmation of clinical APEC isolates

The APEC strains were isolated by inoculating swabs on MacConkey agar (Himedia, India) and blood agar media consisting of 5% sheep’s blood in Mueller Hinton agar (Himedia). The cultured samples were incubated overnight at 37°C. The *E. coli* isolates were identified based on their characteristics, conventional biochemical tests, and the API 20E test (BioMerieux, France). A single colony was resuspended in 300 μL of sterile ultrapure water and subcultured in Muller Hinton broth (MHB, Himedia) for further antimicrobial susceptibility testing. The remaining suspended bacteria were used for DNA isolation, as described by Martins *et al*. [19]. Briefly, the suspended bacteria were boiled in ultrapure water at 100°C for 10 min and centrifuged at 10,000× *g* for 5 min. The supernatant was used as a template for identifying the *E. coli* strain, as described by Chen and Griffiths [20]. Briefly, the primers (forward: 5’-CCGATACGCTGCCAATCAGT-3’ and reverse: 5’-ACGCAGACCGTAGGCCAGAT-3’) derived from nucleotide sequences flanking the gene encoding the universal stress protein (*uspA*) were used to amplify an 884-base pair (bp) specific product, confirming the *E. coli* strain. The polymerase chain reaction was performed using a Biometra thermal cycler (Prague, Czech Republic) with the PCR Master Mix (2X; Thermo Fisher Scientific). The DNA template was preheated at 94°C for 5 min and then amplified for 30 cycles, each consisting of 94°C for 2 min, 70°C for 1 min, and 72°C for 1 min. The amplified product was visualized using a 1.5% agarose gel in 1× Tris-borate-EDTA buffer at 100 V, followed by staining in ethidium bromide (1 µg/mL). The confirmed *E. coli* isolates were further used for the antimicrobial sensitivity test.

### Minimum inhibitory concentration (MIC)

Pure standard colistin was obtained from the DANA pharmaceutical factory for veterinary products (Nablus, Palestine). Colistin was diluted in 10% acetic acid to prepare a stock solution (10 mg/mL). The reference *E. coli* strain (ATCC 25922) was tested using colistin to check the effectiveness and to rule out solvent inhibitory effect. Each new colistin stock solution was retested using this strain (ATCC 25922) to ensure reproducibility. A susceptible breakpoint of ≤2 μg/mL and a resistant breakpoint of ≥4 μg/mL were considered according to The European Committee on Antimicrobial Susceptibility Testing [21]. The susceptibility of APEC isolates toward colistin was evaluated using broth microdilution with 96-well microplates (Eppendorf, Hamburg, Germany). For testing the field APEC isolates, colistin was used at concentrations ranging between 2 and 64 μg/mL. To prepare the bacterial inoculum, a suspension of 0.5 McFarland standard (0.5 mL of 0.048 M BaCl_2_ to 99.5 mL of 0.18 M H_2_SO_4_) was prepared [22]. The density of the APEC bacteria was adjusted to 0.5 McFarland standard using sterile distilled water within a concentration range of 10^7^–10^8^ colony forming units (CFU)/mL. The bacterial suspension was further diluted 100× using MHB to 10^6^ CFU/mL. Finally, 100 μL of antimicrobial was added to the first column and 50 μL of MHB to the other wells, followed by a twofold serial dilution. Colistin was applied to all samples at 2–64 μg/mL concentrations. The contents of the well were adjusted to 100 μL by adding 50 μL of the bacterial inoculum (1 × 10^6^ CFU/mL) to reach a final concentration of 5 × 10^5^ CFU/mL. Non-inoculated MHB was included as a negative control. The MIC against colistin was tested for all APEC isolates in triplicate. The plates were incubated aerobically at 37°C overnight.

### Detection of mobile colistin resistance (*mcr*-1) gene

The DNA extracted from the colistin-resistant APEC isolates was used to detect the *mcr-1* gene, as described by Gharaibeh *et al*. [23]. Briefly, the primers (forward: 5’-AGTCCGTTTGTTCTTGTGGC-3’ and reverse: 5’-AGATCCTTGGTCTCGGCTTG-3’) targeting *mcr-1* were used, which produced a 320 bp amplicon. The DNA from the reference *E. coli* strain NCTC 13846 (*mcr*-1 positive) was used as the positive control. Each PCR reaction consisted of 12.5 μL PCR Master Mix (2X; Thermo Fisher Scientific), 8.5 μL nuclease-free water, 0.5 μL of each primer (10 μM), and 3 μL DNA template. The cycling conditions were: One cycle of denaturation at 94°C for 15 min followed by 25 cycles, each consisting of 94°C for 30 s, 58°C for 90 s, 72°C for 60 s, and a final cycle at 72°C for 10 min. The amplified product was visualized using a 1.5% agarose gel at 100 V and then stained using ethidium bromide (1 µg/mL).

### Statistical analysis

Descriptive statistics were performed for the MIC values toward colistin and the presence of *mcr-1* among colistin-resistant isolates. The relationship between the age of the bird, region, or period and the presence of resistance was compared using Pearson’s Chi-squared test using SPSS version 22^®^ (IBM, New York, USA). The figures were generated using GraphPad Prism^®^ 5.0 (GraphPad Software, Inc., La Jolla, CA, USA).

## Results

**Figure-1:**
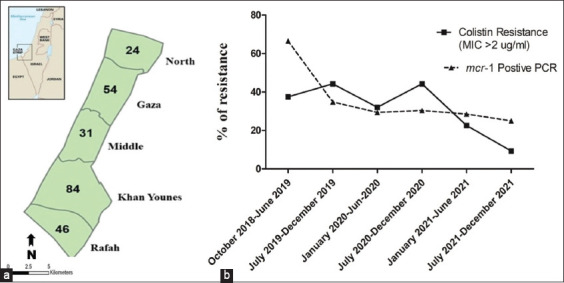
Avian pathogenic *Escherichia coli* (APEC) samples tested using minimum inhibitory concentration toward colistin between October 2018 and December 2021. (a) Distribution of samples according to the region in Gaza Strip. (b) Colistin resistance of APEC isolates (solid line) and abundance of *mcr*-1 gene (dash line) in the study period within 6 months intervals.

**Figure-2:**
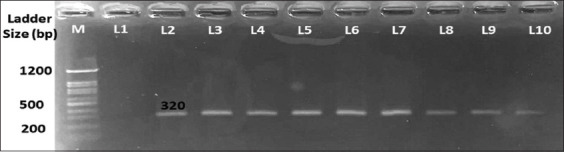
Agarose gel electrophoresis of mobile colistin resistance gene (*mcr-1)* polymerase chain reaction products from colistin resistance avian pathogenic *Escherichia coli* isolates from turkeys’ clinical cases. Positive isolates (Lanes 3–10) show the expected band size (320 bp). Lane 1 represents the DNA *E. coli* reference strain (ATCC 25922) as a negative control. Lane 2: DNA of the *E. coli* reference strain NCTC 13846 (*mcr*-1 positive) as a positive control.

The MIC of the reference *E. coli* strain (ATCC 25922) was 0.5 μg/mL, which is within the expected range [22]. We obtained 239 APEC clinical isolates from birds that exhibit typical pathological lesions, including pericarditis, perihepatitis, and peritonitis. Colistin resistance was observed in 32.2% (n = 77) of the isolates (MIC ≥ 4 μg/mL). Colistin-resistant APEC was observed in all regions and ages during all 6-month intervals of the study period. No significant difference was observed in the percentage of colistin resistance of APEC isolates based on the area and age (Table-1). The number of resistant APEC isolates was significantly higher between October 2018 and June 2019 (37.5%). However, it was significantly lower between July 2021 and December 2021 (9.3%) (p < 0.001) (Figure-1). The colistin-resistant APEC isolates were further analyzed using PCR to detect *mcr-1*. The 320 bp amplicons of the target *mcr-1* gene were detected as described by Gharaibeh *et al*. [23]. Out of 77 resistant *E. coli* isolates, 25 were *mcr-1*-positive, representing 10.5% of the total APEC isolates (n = 239) and 32.5% of phenotypic colistin resistance isolates (n = 77) (Figure-2).

**Table-1:** The risk factors associated with the prevalence of colistin resistance and *mcr-1* gene in avian pathogenic *Escherichia coli* collected from clinical turkey cases in Gaza Strip.

Variable	Number of samples (% Resistance)	*mcr-1*/Colistin resistance APEC (%)
Location		
North	24 (33.3)^a^	2 (25.0)^a^
Gaza	54 (33.3)^a^	2 (11.1)^a^
Middle	31 (19.4)^a^	3 (50.0)^a^
Khan Younes	84 (32.1)^a^	6 (22.2)^a^
Rafah	46 (39.1)^a^	12 (66.7)^a^
Age (Days)		
<40	146 (29.4)^a^	16 (37.2)^a^
40–80	75 (34.7)^a^	6 (23.1)^a^
>80	18 (44.4)^a^	3 (37.5)^a^
Period (6 months)		
October 2018–June 2019	8 (37.5)^a^	2 (66.6)^a^
July 2019–December 2019	52 (44.2)^b^	8 (34.8)^a^
January 2020–June 2020	53 (32.1)^a^	5 (29.4)^a^
July 2020–December 2020	52 (44.2)^b^	7(30.4)^a^
January 2021–June 2021	31 (22.6)^a^	2 (28.5)^a^
July 2021-December 2021	43 (9.3)^b^	1 (25.0)^a^

Each superscript letter denotes a subset of categories whose column proportions do not significantly differ from each other at the 0.05 level, mcr-1=Mobile colistin resistance gene

## Discussion

In humans, colistin is used as a last-resort antimicrobial agent to treat infections caused by multidrug-resistant Gram-negative organisms, such as carbapenemase-producing *Enterobacterales*, *Acinetobacter baumannii*, and *Pseudomonas aeruginosa* [24, 25]. The intensive rearing practices in the turkey meat industry in the Gaza Strip cause increased economic losses due to diseases such as colibacillosis. Poultry farmers in Gaza Strip usually administer colistin through water or as off-label injections without veterinary supervision, even in the absence of disease symptoms. Antibiotic use in farm animals has received little attention, particularly in developing countries, including Palestine, increasing the threat of AMR [26]. In the Gaza Strip, phenotypic resistance toward colistin was previously reported in 41% of the *Enterobacteriaceae* clinical isolates obtained from human microbiological laboratories [27] and 14.5% of chicken fecal materials [28]. Our study detected a high overall prevalence (37.5%) of colistin-resistant APEC strains in the clinical samples from turkeys. Colistin resistance was detected in all investigated areas and during different ages of the birds, indicating the continuous prevalence of colistin resistance in Gaza Strip. Despite the difference in the study design and the host species, our findings suggest a higher prevalence of colistin resistance among APEC than previously reported. In France, 0.5%, 1.8%, and 5.9% colistin-resistant *E. coli* isolates were found in samples isolated from pigs, poultry, and turkeys, respectively [29]. The highest number of phenotypically colistin-resistant *E. coli* samples expressing *mcr-1* was reported in China (in 20% of the isolates) [4]. Among the 41 colistin-resistant *E. coli* isolates reported in Nepal, 18 (43.9%) were positive for the plasmid-mediated *mcr-1* gene [30]. In Lebanon, 98% of *E. coli* isolates from poultry were *mcr-1-*positive [31]. Plasmid-mediated colistin resistance commonly affects the food chain worldwide [32]. Our study is the first to describe the presence of the *mcr-1* gene among *E. coli* isolated from chronic respiratory cases in turkey flocks in the Gaza Strip. Approximately 10.5% of APEC isolates were *mcr*-1-positive, representing 32.5% of all colistin-resistant isolates. This suggests that other *mcr* genes might be circulating in Gaza Strip. Moreover, multiple occurrences of *mcr* genes have been reported in *E. coli* [33, 34]. One of the limitations of this study was the lack of screening for other *mcr* genes. Further research is required to understand the prevalence of organisms with antibiotic resistance patterns in poultry to provide a broader perspective on the AMR pattern of poultry organisms. This might help reduce the chances of infection with these resistant strains in humans.

Our findings showed a significant decrease in colistin resistance among APEC in the past 6 months of the study, possibly due to the ban on colistin use for livestock in Gaza Strip in December 2018. Further and continuous follow-up of colistin resistance is needed to evaluate the efficacy of this ban on the prevalence of colistin resistance. Similarly, colistin resistance and *mcr*-1 prevalence among *E. coli* in pigs reportedly decreased immediately after the ban [35]. In China, the ban on using colistin as a growth promoter in livestock (pig, chicken, and cattle) significantly reduced colistin resistance and the prevalence of *mcr-1* in both animals and humans [36]. However, these studies also recommend that colistin resistance and the presence of *mcr* genes should be continuously monitored in humans and food-producing animals.

## Conclusion

Our results prove that colistin resistance in APEC causing clinical diseases in turkeys is a significant health concern in Gaza Strip. The ban on colistin use in livestock in Gaza Strip reduced colistin resistance and *mcr*-1 abundance among clinical APEC isolates in turkeys. However, further studies and continuous monitoring of antimicrobials are required in humans and food-producing animals.

## Author’s Contributions

All authors contributed to the study’s conception and design. AMT, IMA, NAA, AFF, BYAH, NHJ, and MHG: Material preparation, data collection, clinical and laboratory work, and analysis. IMA and AMT: The first draft of the manuscript. All authors have read, reviewed, and approved the final manuscript.
